# Supplementary material for: Analysis of Gut Bacterial and Fungal Microbiota in Children with Autism Spectrum Disorder and Their Non-Autistic Siblings
Source: Nutrients. 2024 Sep 5;16(17):3004. doi: 10.3390/nu16173004 (PMC11396985; doi:10.3390/nu16173004)
Supplement: Supplementary file 1 [file nutrients-16-03004-s001.zip › Supplemental Tables1-5.pdf]

Supplemental Table 1. Organisms (bacteria and yeast) screened for their ability to break down fibers

Bifidobacterium animalis	*Lactobacillus paracasei*
Bifidobacterium bifidum	*Lactobacillus plantarum*
Bifidobacterium breve	*Lactobacillus reuteri*
Bifidobacterium lactis	*Lactobacillus rhamnosus*
Bifidobacterium longum subps. infantis	*Lactobacillus salivarius*
Bifidobacterium longum	*Lactococcus lactis*
Lactobacillus acidophilus	*Pediococcus acidilactici*
Lactobacillus casei	*Pediococcus pentosaceus*
Lactobacillus delbrueckii subsp. bulgaricus	*Saccharomyces boulardii*
Lactobacillus delbrueckii subsp. lactis	*Streptococcus thermophilus*
Lactobacillus gasseri	

**Supplemental Table 2.** Demographic Information for Enrolled subjects	
**Factor**	**Frequency (n=76)**
Age at diagnosis: <1/1/2/3	11/24/34/7
Race (Asian/Black/Indonesian/White)	9/2/2/63
GIT up (no/yes)(2 missing)	58/16
GIT low (no/yes)	34/42
Breast feeding (no/yes)	14/62
Antibiotic before age 4 (6 missing)	7/63
Sex (female/male)	22/54
C-section (no/yes)	54/22
Infection during pregnancy (no/yes)	50/51
Proton Pump Inhibitors no/yes (1 missing)	65/10
Antibiotics before 6 months (no/yes)	50/26
Probiotics (1 missing)(no/yes)	40/35
Seizure (6 missing)	54/16
**Factor**	**Mean (standard deviation "SD")**
Exercise	5.2 (2.3)
Number of antibiotics	6.8 (7.8)
Processed food	7.2 (4.9)
Red meat	2.6 (2.4)
Sweets	5.5 (4.2)
Vegetables	8.0 (4.9)
Fish	0.7 (0.9)
Fruit	9.7 (6.5)
Chicken	4.6 (3.6)
Beans	2.9 (3.7)
Whole grain	7.6 (4.8)
Body mass index	19.3 (4.6)
How long from prior bowel movement (hrs)	3.1 (1.1)
Age (years) at data collection: mean (STD)	11.5 (6.5)

**Supplemental Table 3.** Univariate analysis of Microbiome data (factors that were statistically significantly associated with autism		
**Factors (per percentage of abundance increase if not specified)**	**Odds ratio**	**p value**
p__Cyanobacteria (**per 0.01 percentage of abundance increase)**	0.81	0.01
c__Chloroplast (**per 0.01 percentage of abundance increase)**)	0.75	0.02
s__Prevotella nigrescen (**per 0.01 percentage of abundance increase**)	0.70	0.06
g__Anaerostipes	0.31	0.06
g__Bacteroides	0.98	0.06
o__Streptophyta (**per 0.01 percentage of abundance increase**)	0.78	0.07
o__Burkholderiales	1.97	0.07
g__Brevundimonas (**per 0.01 percentage of abundance increase**)	9.4	0.07
o__Boletales	0.39	0.07
c__Betaproteobacteria	1.82	0.08
c__Chytridiomycetes (**per 0.01 percentage of abundance increase**)	0.78	0.08
f__Chromatiaceae (**per 0.01 percentage of abundance increase**)	4.0	0.08
s__Galactomyces_geotrichum	0.99	0.08
s__Coprococcus eutactus	0.45	0.08
g__Geotrichum	0.77	0.08
g__Trabulsiella (**per 0.01 percentage of abundance increase)**	1.16	0.08
f__Clavicipitaceae	1.72	0.09
f__Trichosporonaceae	0.96	0.09
g__Leptothrix (**per 0.01 percentage of abundance increase**)	1.85	0.09
f__Bacteroidaceae	0.97	0.09
p__Chytridiomycota (**per 0.01 percentage of abundance increase)**	0.88	0.09
g__Shewanella (**per 0.01 percentage of abundance increase)**	1.14	0.09
f__Alcaligenaceae	2.15	0.09
g__Delftia (**per 0.01 percentage of abundance increase)**	1.5	0.09
g__Azospirillum (**per 0.01 percentage of abundance increase)**	0.67	0.09
f__Marasmiaceae	3.45	0.10
c__Spirochaetes (**per 0.01 percentage of abundance increase)**	0.76	0.10
g__Metarhizium	16.85	0.10

Supplemental Table 4. Univariate analysis of survey data (factors that are statistically significantly associated with autism).

factors	Odds ratio	p value
Sex (male VS female)	8.38	< 0.001
Seizure (Yes VS No)	5.60	0.03
upper gastrointestinal disturbances (Yes VS No)	4.94	0.05

Supplemental Table 5. Comparison of Concordance index of models built from different strategies.

Models	C-index
Model 1: Important bacteria and fungi (at taxonomic level of 2, 6, 7) selected using screening and LASSO	0.935
Model 2: Important demographic, diet factors selected using screening and LASSO	0.865
**Model 3:** Important bacteria and fungi (at taxonomic level of 2, 6, 7) and demographic, diet factors selected using screening and LASSO	0.983
Model 4: Important bacteria and fungi (at all taxonomic level) and demographic, diet factors selected using screening and LASSO	0.956
